# *In Vitro* Antioxidant and Antimicrobial Potency of *Mimosa pudica* of Nepalese Terai Region: Insight into L-Mimosine as an Antibacterial Agent

**DOI:** 10.1155/2022/6790314

**Published:** 2022-10-07

**Authors:** Ashok Kumar Mandal, Anisha Pandey, Ranjit Kumar Sah, Adesh Baral, Phoolgen Sah

**Affiliations:** ^1^Natural Product Research Laboratory, Thapathali, Kathmandu 44600, Nepal; ^2^Department of Biotechnology, National College, Tribhuvan University, Naya Bazar, Kathmandu 44600, Nepal; ^3^Department of Pharmacy, Janamaitri Foundation Institute of Health Sciences, GPO Box No. 8322, Lalitpur, Nepal; ^4^Department of Infection and Immunology, Kathmandu Research Institute for Biological Sciences, Biomedicum Research Campus Building, Saptakhel, Lalitpur 44700, Nepal

## Abstract

**Aim:**

The study aimed to evaluate the in vitro antioxidant and antimicrobial potency of *Mimosa pudica* found wildly in the Terai region of Nepal and assess its physicochemical properties, such as total phenolic content (TPC) and total flavonoid content (TFC).

**Materials and Methods:**

The physicochemical properties of ethyl acetate extract of *Mimosa pudica* (EAMP), such as extractive value, total ash content, loss on drying, and phytochemical screening, were calculated using standard protocols. The TPC was determined by using the Folin–Ciocalteu method taking gallic acid as standard, and TFC was conducted by using the AlCl_3_ colorimetric method, using a 96-well plate reader. The in vitro antibacterial activity of different concentrations of the extract against four bacterial ATCC strains was determined by the agar well diffusion method in the Mueller Hinton agar (MHA) medium. The in silico molecular docking model was used to ascertain the antibacterial potency of L-mimosine against the selected strains of bacteria used for the in vitro study by calculating the binding affinity towards the protein of bacteria.

**Results:**

The preliminary screening of the extract showed the presence of several phytochemicals. The total ash content (7.67%), loss on drying (2.30%), and extractive value (8.966%) were determined by analyzing the crude sample. The total phenolic and flavonoid contents were 418.640 ± 0.018 mg GAE/g (dried extract) and 14.126 ± 0.021 mg QE/g (dried extract), respectively. The extract showed a potent free radical scavenging activity with an IC50 value of 158.95 ± 1.12 *µ*g/mL. The plant extract also demonstrated the antibacterial activity against both Gram-positive bacteria *Staphylococcus aureus* (15 mm) and *Bacillus cereus* (22 mm) and Gram-negative bacteria *Escherichia coli* (17 mm) and *Klebsiella pneumoniae* (16 mm) at 200 mg/mL concentration of extract. There was a noteworthy binding affinity of antibiotics with almost all selected bacterial proteins with binding energy against *Escherichia coli* DNA gyrase subunit B (−5.7 kcal/mol), *Staphylococcus aureus* DNA gyrase subunit B (−6.1 kcal/mol), *Bacillus cereus* metallothiol transferase (−5.2 kcal/mol), and *Klebsiella pneumoniae*beta-lactamase (−6.1 kcal/mole), respectively, with the L-mimosine.

**Conclusion:**

The findings of the current study suggest that *Mimosa pudica* from the Terai region of Nepal is rich in phenolic and flavonoid compounds, has a significant impact on bacterial growth inhibition, and has a notable potential to scavenge free radicals (DPPH). According to the in silico analysis, L-mimosine is a potent antibacterial compound that might be utilised to discover novel antibacterial drugs to combat antibiotic resistance.

## 1. Introduction

Nature has been a source of medicine for thousands of years. The phytochemical constituents and the potency of plants against several diseases attracted scientists to explore natural resources for discovering novel drugs. WHO has reported that 80% of the world population still relies on traditional medicine for primary health care [1]. Scientists have recognized the necessity to screen plants to find innovative drugs with therapeutic efficacy in the current context of multiple medication resistance. *Mimosa pudica* (Family: Fabaceae), a neglected weed, has been studied for its numerous ethnobotanical uses. It is a perennial or annual creeping herb and has been identified as Lajjalu in Ayurveda. It is one of the sought-after plants for its pharmacological properties, which include antidiabetic, antitoxin, antihepatotoxin, antioxidant, and wound-healing properties [2]. The plant has been widely mentioned in Ayurveda and the Unani medicine system. In the past, and still in several parts of the world, the different parts of *Mimosa* are used to relieve several illnesses and health discomfort. The root decoction of this plant is used to relieve toothache. *Mimosa pudica* is reported to stop the bleeding and speed up the healing of the wound. It is mostly utilised in herbal remedies for gynecological conditions [3].

The phytochemicals of plants are attributed to multiple pharmacological activities, and they can be screened with some in vitro assays believing that they have the same in vivo potency. Free radicals are highly reactive moieties produced by cells during respiration and cell-mediated immunological responses. Free radicals, such as unpaired electrons or reactive oxygen species (ROS), which contain different oxygen species, such as hydrogen peroxide, can cause a variety of damages, eventually leading to the development of multiple human health disorders, including cancer. Antioxidants, being molecules that impede the oxidation of other molecules by donating electrons that can neutralize radical production can alleviate ROS-induced cell damage. The secondary metabolites such as polyphenol and flavonoids with at least one hydroxyl group exhibit free radical inhibition, peroxide breakdown, metal inactivation, and oxygen scavenging and play a critical role in multiple biological activities [4, 5]. In biological systems, some phenolic compounds promote the cellular synthesis of endogenous antioxidants [6]. The presence of different phytochemicals, such as carbohydrates, terpenoids, phenols, aliphatic, and aromatic molecules, peptides, is supposed to be responsible for the antimicrobial activity of medicinal plants [7]. Antibiotic and multidrug-resistant bacteria are currently confronting challenges for researchers in drug discovery and health care across the world. Early isolates revealed a significant genetic component responsible for resistance [8]. New antimicrobial chemicals are desperately needed. As a result, researchers are increasingly turning to ethnomedicine in quest of novel leads to develop better medications against microbial diseases. The phytochemical exploration of medicinal plants is not only for the identification of bioactive compounds but also for revealing new sources of economic phytocompounds for the synthesis of complex chemical substances, as well as for determining the true relevance of traditional medicines. The objective of this study is to investigate the phytocompounds present in the *Mimosa pudica*, estimate the phenolic and flavonoid content, and assess the in vitro antioxidant and antimicrobial effectiveness against gram-positive and negative bacterial strains. Furthermore, A molecular docking model was used to ascertain the affinity of L-mimosine for bacterial protein and to assess the antimicrobial property of the compound.

## 2. Methodology

### 2.1. Plant Collection and Authentication

The leaves of *M. pudica* were collected in October 2021 from Haripurwa Municipality located in Sarlahi district of province no. 2 of Nepal. The herbarium of the plant was prepared successfully, and the plant was identified and certified by the National Herbarium and Plant Laboratory (KATH), Godawari, Lalitpur, and a voucher specimen (06/078–079) was deposited.

### 2.2. Extraction

The maceration process was employed for extraction of leaf samples using ethyl acetate as a solvent. Firstly, the collected leaves of *M. pudica* were kept in the shade and dried for a week and then the leaves were grounded into a powder. The powdered sample was subjected to the first maceration in ethyl acetate for 2 days, followed by the second maceration for another 2 days. The extract from successive maceration was then filtered and concentrated using the Rota-evaporator. Thus, the obtained extract was kept inside the vacuum desiccators for complete loss of water and finally transferred in a bottle, labeled, and stored in a refrigerator.

### 2.3. Phytochemical Screening

The dried extract of the sample then proceeded for phytochemical screening and solvent-solvent fractionation was conducted using hexane, ethyl acetate, and water to identify the constituents, using standard phytochemical methods as described by Trease and Evans as well as Harborne [9, 10]. The screening involves the detection of carbohydrates, proteins, amino acids, phytosterol, triterpenoids, tannins, flavonoids, alkaloids, fixed oil, fats, saponin, and glycosides.

### 2.4. Physicochemical Analysis

The leaf sample was grounded and physicochemical parameters (extractive value, total ash content, and loss on drying) were analyzed using the standard quality control guideline of WHO [11].

### 2.5. Determination of Total Phenolic Content

The total phenolic content of *Mimosa pudica* was determined using the Folin–Ciocalteu method, taking gallic acid as standard for the calibration curve as described by Singleton et al. with a few modifications [12]. Briefly, 2 *µ*l extract (5 mg/mL) was pipetted in a triplicate manner and was then mixed with 158 *µ*L distilled water and 10 *µ*L of 10% w/v Folin–Ciocalteu reagent (FCR) in a 96-well plate. The mixture was then left for 8 minutes at room temperature, and the initial reading was taken at 765 nm. Subsequently, after that, 30 *µ*L of 20% (w/v) Na_2_Co_3_ was added to each well and was incubated for 30 min at 40°C. The final absorbance was taken at 765 nm using a 96-well plate reader (EpochTM 2 Microplate Spectrophotometer, BioTek Instruments, USA). The amount of total phenolic content was calculated using a gallic acid calibration curve and reported as gallic acid equivalent in mg per gram of dry extract (mg GAE/g DE).

### 2.6. Determination of Total Flavonoid Content

The flavonoid content in *M. pudica* extract was quantified using a 96-well plate reader employing the Dowd technique with a little modification [13]. An aliquot of 20 *µ*l of extract solution (5 mg/ml) was mixed with 110 *µ*l distilled water and 60 *µ*l methanol was pipetted in a well. The solution was then kept shaking for 5 min to let the reaction happen. The initial reading was taken at 415 nm. The reaction was then quenched by adding 5 *µ*L of 10% AlCl_3_ and 5 *µ*L CH_3_COOK (1M) and was incubated for 30 min at 25°C. The final reading was taken after incubation at 415 nm. Quercetin standard (0–100 ppm) was used to prepare the calibration curve. The total flavonoid content was calculated and expressed as mg quercetin equivalent per gram of dry extract (mg QE/g DE).

### 2.7. Antioxidant Property: DPPH Scavenging Assay

In vitro antioxidant activity of the extract of *Mimosa pudica* leaves was determined using the DPPH free radical scavenging assay method described by Brand-Williams et al. with a little modification [14]. To do this assay, 100 *µ*L extract or reference quercetin of different concentrations were pipetted in triplicate in a 96-well plate, initial reading was taken at 517 nm, and 100 *µ*L of 0.1 mM DPPH was added to each well and incubated at room temperature for 30 min in the dark. A control was prepared by mixing 100 *µ*L methanol and 100 *µ*L DPPH solution. Finally, the absorbance of the solutions was measured by using a 96-well plate reader at 517 nm. The percentage of inhibition was calculated by using the following formula:

(1) $$\begin{array}{c} \%\text{Inhibition}=\left(\frac{A_{\text{control}}-A_{\text{sample}}}{A_{\text{control}}}\right)\times 100, \end{array}$$

where A _control_ is the absorbance of the control and A _sample_ is the absorbance of the sample.

### 2.8. Antibacterial Screening of Extracts

Antibacterial activity was evaluated according to the method described by Satish et.al. and Joshi et al. [15]. Antibacterial activity of ethyl acetate extract of *Mimosa pudica* was determined by the agar well diffusion method in Mueller Hinton agar (MHA) medium. The ATCC cultures of bacteria, namely, *Staphylococcus aureus* (ATCC 6538 P), *Bacillus cereus* (ATCC 14579), *Escherichia coli* (ATCC 8739), and *Klebsiella pneumoniae* (ATCC 700603), were taken for the antibacterial test. The selected bacterial strains were cultured overnight and used to assess the inhibitory potency of plant extract. The bacteria were first tallied with 0.5 MacFarland solution. Wells were made of agar plates using a cork borer of 6 mm diameter. Then, 100uL of each extract was placed in the wells made of the inoculated plates; the process also included the same volume of ethyl acetate, which served as the negative control. Likewise, for positive control, three antibiotics (gentamycin, clotrimazole, and cefotaxime) were taken. All the plates were incubated for 24 hours at 37°C. The zone of inhibition (ZOI) was measured the following day.

### 2.9. Systematic Review of L-Mimosine in the Ethyl Acetate Extract of *Mimosa pudica* (EAMP)

A detailed systematic literature review was conducted for the exploration of L-mimosine as the one of constituents found in ethyl acetate extract of *Mimosa pudica*. Research and review papers published in several databases (PubMed, Scopus, Google Scholar, Web of Science) were explored to collect information. The relevant keywords having an entry on medical subject headings (MeSH) database, and which are already indexed, were used as the precursor for backward and forward search strategy. The articles not complying with the review protocol were excluded.

### 2.10. In Silico Analysis of Antibacterial Potency by Molecular Docking

Docking of L-mimosine (PubChem Id: 440473) was conducted in four protein structures selected from four different bacteria . DNA gyrase subunit B (PDB: 1KZN) was selected for *Escherichia coli*, and DNA gyrase subunit B (PDB:4uro) was selected for *Staphylococcus aureus,* metallothiol transferase (PDB: 4jh5) was selected for *Bacillus cereus,* and beta-lactamase (PDB: 4pm9) was selected for *Klebsiella pneumoniae.*

For positive control, four different antibiotics were used, clorobiocin (PubChem Id: 54706138), novobiocin (PubChem Id: 54675769), fosfomycin (PubChem Id: 446987), and cefotaxime (PubChem Id: 5742673) against *Escherichia coli, Staphylococcus aureus, Bacillus cereus, and Klebsiella pneumoniae* selected proteins, respectively.

For the preparation of the protein, the AutoDock tool's graphic interface was used. First, the water molecules were removed, then the addition of polar hydrogen was conducted after that nonpolar hydrogen was merged, and finally, the Kollman charge was added [16].

In the case of ligands, SDF files were converted to pdbqt files, and energy minimization was conducted by applying Universal Force Filed in an Open Babel (Open Babel Development Team) software [17].

Docking of ligands to the protein active site was performed in AutoDock and Vina [18] conjugated with PyRx software Dallakian. [19]. The docking parameter file and grid parameter file were set, setting the active site residue at the center of the grid box, and the grid points for auto grid calculation were set as 25 × 25 × 25 Å. The Lamarckian genetic algorithm was used in the overall process. This algorithm was used for calculating protein-fixedligand-flexible calculations [20].

Pymol software was used for visualization of the 3D structure of docked files and the ligPlot+ (European Bioinformatics Institute) software [21] was for visualization of the 2D image of the interacting atoms between ligands and proteins.

## 3. Result and Discussion

### 3.1. Phytochemical Screening

The investigations during phytochemical screening of ethyl acetate extract of *Mimosa pudica* (EAMP) showed the presence of flavonoids, saponins, coumarins, tannins, terpenoids, and alkaloids (Table 1). The preliminary phytochemical screening carried out by Ittiyavirah and Pullochal revealed the presence of alkaloids, flavonoids, phenolics, and tannins in the ethanolic extract of *Mimosa pudica* whole plant [22].

### 3.2. Physicochemical Analysis

The physicochemical analysis of the crude powder of *Mimosa pudica* leaves was conducted and found noteworthy. The analysis result is well implicated through Table 2. Physicochemical standards have great significance in authentication and assuring quality, purity, and thereby, medicinal efficacy of the medicinal plants [23]. Thus, the comparative data matching and noteworthy results make *Mimosa pudica* a choice of plant with therapeutic potency.

### 3.3. Total Phenolic Content

**Figure 1:**
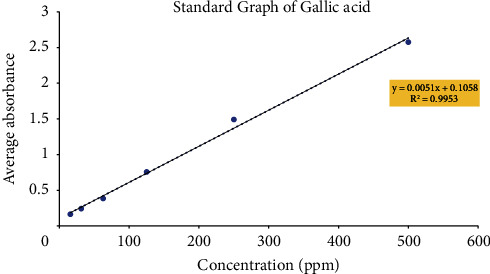
Standard calibration curve of gallic acid for determination of total phenolic content.

The phenolic content of the EAMP was determined in the term of gallic acid equivalent (GAE). The hydroxyl groups in the phenolic compounds of plant extracts are vital with redox properties responsible for antioxidant activity channelizing by facilitating free radical scavenging [24]. As a basis, phenolic content was measured using the Folin–Ciocalteu reagent in extract. The results were derived from a calibration curve (*y* = 0.0051*x* + 0.1058, *R*^2^ = 0.9953) of gallic acid (Figure 1) and expressed in mg gallic acid equivalents (GAE) per gram dry extract (DE). The total phenolic content was found to be 418.640 ± 0.018 mg GAE/g DE (Table 3). Comparing the works from the literature; Jimenez et al. reported a total phenolic content of 323 mg GAE/g in the *Mimosa* genus; i.e., *Mimosa albida* [25]. Patro et al. found a TPC of 15.64 ± 1.31 mg GAE/g in ethyl acetate extract of *Mimosa pudica* leaves [26]. The data of this study vary compared to the literature which may be due to the climatic condition, harvesting, and method of extraction, which may have altered the phenolic content.

### 3.4. Total Flavonoid Content

**Figure 2:**
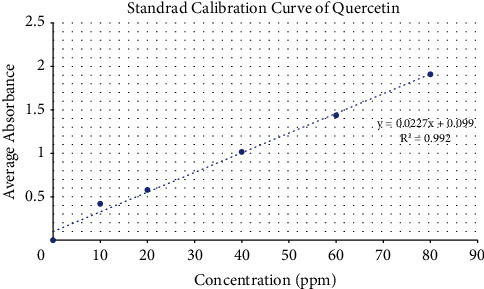
Standard calibration curve of quercetin for total flavonoid content estimation.

The phenolic content of the ethyl acetate extract of *Mimosa pudica* (EAMP) was determined in the term of quercetin equivalent (QE). As a basis for the quantitative determination, flavonoid contents in *Mimosa* extracts were determined using aluminum chloride in a colorimetric method. The results were derived from the calibration curve (*y* = 0.0057 + 0.0127, *R*^2^ = 0.9973) of quercetin (Figure 2) and expressed in quercetin equivalents (QE) per gram dry extract weight. Total flavonoid content was found to be 14.126 ± 0.021 mg QE/g DE (Table 4). Flavonoids are secondary metabolites possessing antioxidant properties, the endurance of which is determined by the number and position of free OH groups. The reports of past studies on *Mimosa pudica* show that Shrestha et al. reported a TFC of 19.747 ± 6.11 mg QE/g and Srishti Prashar et al. determined a TFC of 49.47 mg QE/g in methanolic extract of *M. pudica* [27, 28].

### 3.5. Antioxidant Property: DPPH Scavenging Assay

Knowing that synthetic lab-based antioxidants possess higher side effects, herbal plants are prioritized as a safer and cost-effective alternative to diminishing the free radicals helping in treating several severe complications related to blood vessels, cancer, and hepatitis. DPPH is generally used to assay the free radical scavenging activity of natural antioxidants. Formerly several experiments have reported moderate to strong free radical neutralizing activity of the plant extracts of the Mimosaceae family. DPPH is a light purple colored highly unstable free radical that changes to stable yellow colored after accepting an electron from the plant extract prepared using DMSO. The reaction's extent depends on the antioxidant substances' hydrogen donating potential. The extract showed a free radical scavenging activity with an IC_50_ value of 158.95 ± 1.12 *µ*g/mL. The standard quercetin has an IC_50_ value of 5.23 ± 0.38 *µ*g/mL (Table 5). A plethora of studies conducted on the ethyl acetate extract of *Mimosa pudica* has reported a comparable IC_50_ value to our study. In 2016, Patro et al. found an IC_50_ value of 23.74 *µ*g/mL [26], while Parmar et al. in 2015 found that even a single compound L-mimosine isolated from *Mimosa pudica* shows a vague IC_50_ value of 233.06 *µ*g/mL at a concentration of 250 *µ*M [28]. The variation of the values might be due to the harsh climate of the Terai region and the time of harvesting. As reported in the literature, plants with genetic diversity, environmental, year-to-year variation, and distribution at different geographical locations significantly affect biochemical compositions [29]. The extracted sample of *M. Pudica* can neutralize the DPPH free radicals, showing that the plant extract can be effectively used in scavenging the free radicals produced in the human body from various metabolic activities. This ability converts free radicals to stable neutral, nontoxic compounds and stops the self-occurring free radical chain reaction in the body.

### 3.6. Antimicrobial Assay

The study shows us that the extract has antibacterial activity against Gram-positive bacteria (*Staphylococcus aureus* and *Bacillus cereus*) and Gram-negative bacteria (*Escherichia coli* and *Klebsiella pneumoniae*), and the positive and negative control was taken for standard drug inhibition comparative study. The ethyl acetate solvent was used to prepare the plant extract in five different concentrations (12.5, 25, 50, 100, and 200). As expected, the highest concentration; i.e., 200 mg/ml, showed the maximum inhibition against each bacterial strain (Tables 6 and 7). The highest activity was witnessed against *B. cereus* where 200 mg/ml ethyl acetate concentration of *Mimosa pudica* extract gave the zone of inhibition of 22 mm. Likewise, *S. aureus* was the least inhibited in comparison to other organisms. In the study conducted by (Shrestha et al.) on the same plant extract, the antibacterial activity against *Staphylococcus aureus* and *Bacillus subtilis* was noted. The zone of inhibition (ZOI) given by *Mimosa* extract against these bacteria was found to be 13 mm and 10 mm, respectively [27].

### 3.7. Bioactive Compounds in EAMP through Literature Review

**Figure 3:**
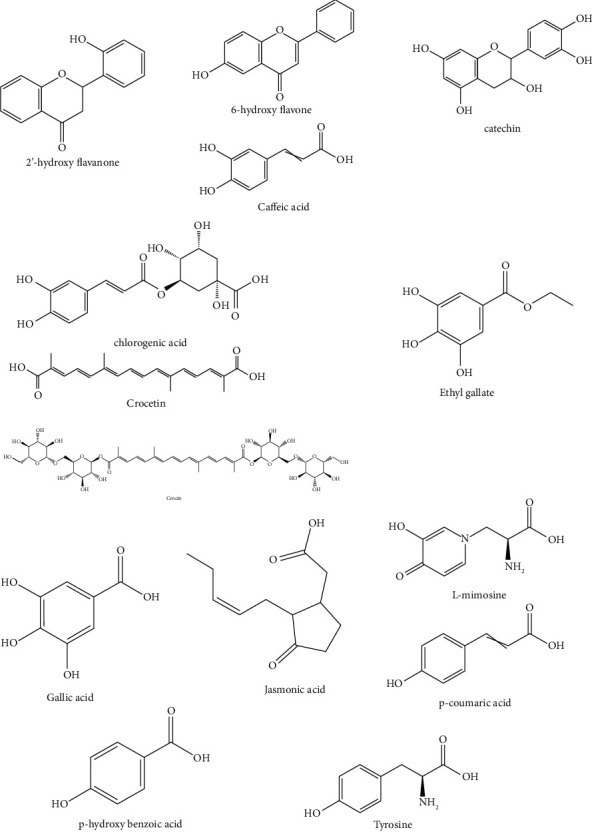
Compounds in ethyl acetate extract of *Mimosa pudica*.

A thorough analysis of the literature found that *Mimosa pudica* contains several marker compounds. The different fractions were found to have varied proportions and composition of constituents [30]. The 14 different chemicals (Figure 3) from the ethyl acetate extract of *Mimosa pudica* were isolated in a study conducted by Patel and Bhutani [31]. The findings of this study showed that the anti-inflammatory effects of *M. pudica* were greatly enhanced by the presence of crocetin, crocin, jasmonic acid, L-mimosine , caffeic acid, ethyl gallate, and gallic acid in the ethyl acetate fraction of the whole plant. This may be partially due to the synergistic effects of these compounds. Moreover, multiple studies have demonstrated that the compound isolated from *Mimosa pudica* has substantial antibacterial, antioxidant, and antidiabetic properties [2]. This study is targeted to gain a better understanding of L-mimosine as an antibacterial chemical through an in silico molecular docking study.

### 3.8. Molecular Docking Study of L-Mimosine for Antibacterial Potency

Four bacteria were used for the antibacterial activity of L-mimosine, binding energies were calculated against their specific protein with specific antibiotic and L-mimosine using molecular docking (Table 8). The proteins for which binding energy was calculated are DNA gyrase subunit B, metallothiol transferase, and beta-lactamase. Antibiotics used were clorobiocin, novobiocin, fosfomycin, cefotaxime, and the -antibacterial compound L-mimosine. The binding energy of *Escherichia coli* DNA gyrase subunit B with clorobiocin and L-mimosine was found to be −9.2 kcal/mol and −5.7 kcal/mol, respectively. Similarly, the binding energy of *Staphylococcus aureus* DNA gyrase subunit B with novobiocin and L-mimosine was found to be −8.2 kcal/mol and −6.1 kcal/mol, respectively. Likewise, the binding energy between *Bacillus cereus* metallothiol transferase with fosfomycin and L-mimosine was −4.3 kcal/mol and −5.2 kcal/mol, respectively, and the binding energy of *Klebsiella pneumoniae*beta-lactamase, cefotaxime, and L-mimosine was found to be −7.5 kcal/mol and −6.1 kcal/mole, respectively.

**Figure 4:**
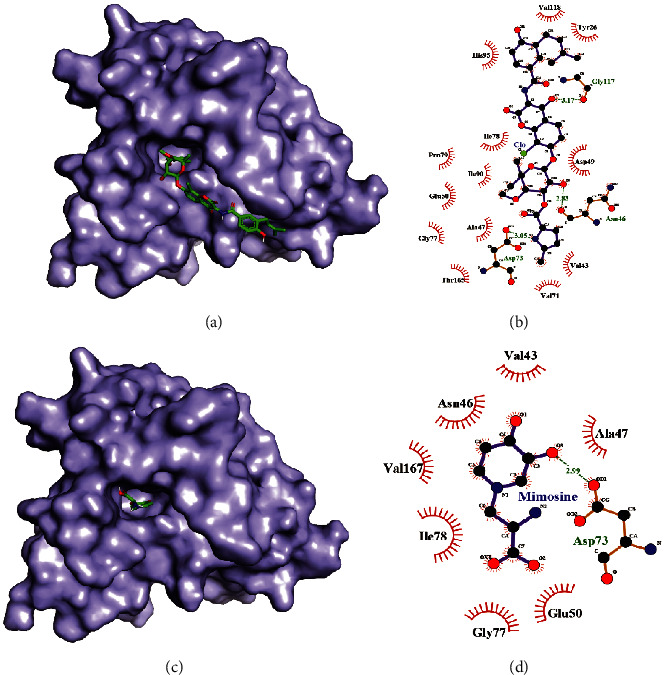
Docked structure of *Escherichia coli* DNA gyrase subunit B with clorobiocin (a) and docked structure of *Escherichia coli* DNA gyrase subunit B with L-mimosine (c). Interacting atoms of DNA gyrase subunit B with clorobiocin (b) and interacting atoms of DNA gyrase subunit B with L-mimosine (d).

Asn46, Asp73, and Gly117 amino acids of *Escherichia coli* DNA gyrase subunit B were involved in forming a hydrogen bond with clorobiocin, whereas Tyr 26, Val43, Ala47, Asp49, Glu 50, Val 71, Gly 77, Ile 78, Pro79, Ile90, His 95, Val118, and Thr 165 amino acids were involved in forming hydrophobic interaction with the clorobiocin molecule (Figures 4(a) and 4(b)). However, only Asp73 was forming a hydrogen bond with the L-mimosine molecule, and Val 43, Asn46, Ala47, Glu 50, Gly77, Ile 78, and Val167 were involved in hydrophobic interaction (Figures 4(c) and 4(d)). There were a greater number of hydrogen bonding and hydrophobic interaction between clorobiocin and the protein than between mimoine and the protein which suggest a greater binding affinity of clorobiocin with *Escherichia coli* DNA gyrase subunit B.

**Figure 5:**
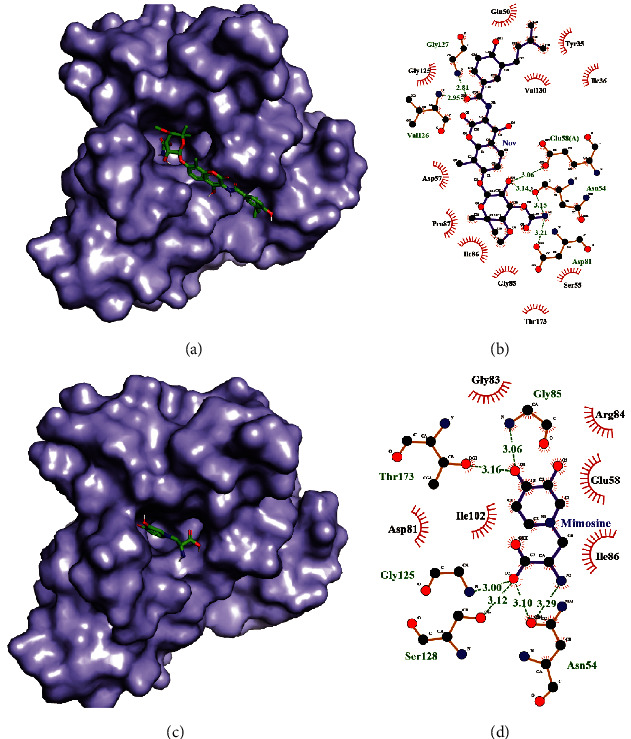
Docked structure of *Staphylococcus aureus* DNA gyrase subunit B with novobiocin (a) and docked structure of *Staphylococcus aureus* DNA gyrase subunit B with L-mimosine (c). Interacting atoms of DNA gyrase subunit B with novobiocin (b) and interacting atoms of DNA gyrase subunit B with L-mimosine (d).

Asn 54, Glu 58, Asp81, Gly 127, and Val 126 amino acids of *Staphylococcus aureus* DNA gyrase subunit B were forming hydrogen bonding with novobiocin and Ile 36, Try 35, Glu 50, Ser55, Asp 57, Gly 85, Ile86, Pro87, Gly 125, Val130, and Thr 173 were forming hydrophobic interaction (Figures 5(a) and 5(b)), whereas Asn 54, Gly 85, Gly125, Ser128, and Thr173 amino acids were forming hydrogen bonding with L-mimosine and Glu 58, Asp81, Gly83, Arg84, Ile86, and Ile102 were informed in hydrophobic interaction with L-mimosine residue (Figures 5(c) and 5(d)). There was similar number of hydrogen bonding, but the number of hydrophobic interactions between novobiocin and the protein was greater than that between mimoine and the protein which suggest a greater binding affinity of novobiocin with *Staphylococcus aureus* DNA gyrase subunit B.

**Figure 6:**
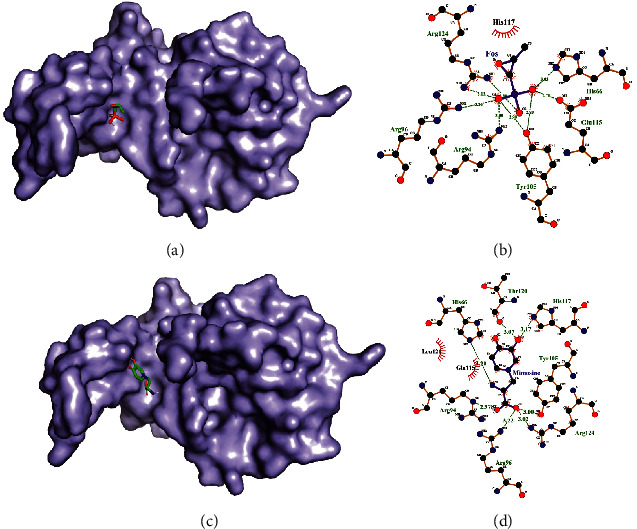
Docked structure of *Bacillus cereus* metallothiol transferase with fosfomycin (a) and docked structure of *Bacillus cereus* Metallothiol transferase with L-mimosine (c). Interacting atoms of metallothiol transferase with fosfomycin (b) and interacting atoms of metallothiol transferase with L-mimosine (d).

His 66, Arg 94, Arg 96, Tyr 105, Glu 115, and Arg 124 amino acids of *Bacillus cereus* metallothiol transferase were forming a hydrogen bond with fosfomycin residue, and His 117 was forming hydrophobic interaction (Figures 6(a) and 6(b)). Meanwhile, His66, Arg94, Arg 96, Arg 124, Tyr 105, His 117, Thr 120, and Glu 115, Leu121 were involved in hydrophobic interaction (Figures 6(c) and 6(d)). Number of hydrogen bonds between fosfomycin residue and the protein were less than that between L-mimosine and the protein and also the number of hydrophobic interactions was greater in L-mimosine and the protein suggesting a greater binding affinity of L-mimosine with *Bacillus cereus* metallothiol transferase.

**Figure 7:**
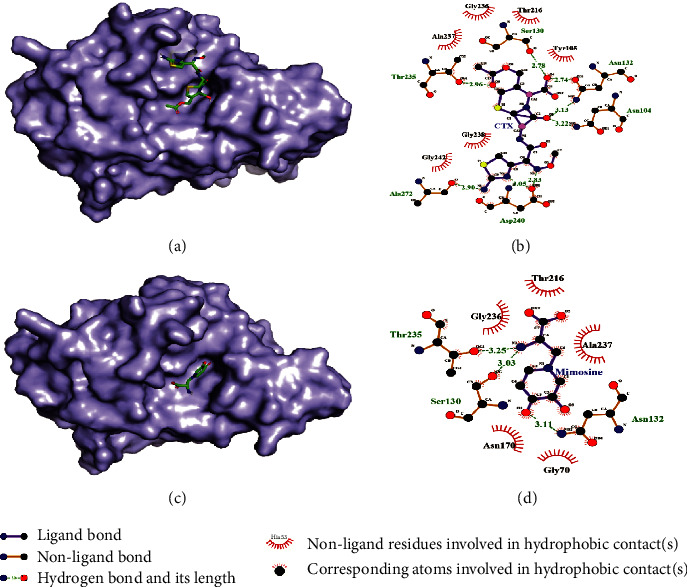
Docked structure of *Klebsiella pneumoniae*beta-lactamase with cefotaxime (a) and docked structure of *Klebsiella pneumoniae*beta-lactamase with L-mimosine (c). Interacting atoms of beta-lactamase with cefotaxime (b) and interacting atoms of beta-lactamase with L-mimosine (d).

Asn 104, Ser 130, Asn 1332, Thr 235, Asp 240, and Ala 272 amino acid residues of *Klebsiella pneumoniae*beta-lactamase were involved in the formation of hydrogen bond with cefotaxime, and Tyr 105, Thr 216, Gly 236, Ala 237, Gly 238, and Gly 242 were forming hydrophobic interaction with cefotaxime (Figures 7(a) and 7(b)). However, only Asn 132, Ser 130, and Thr 325 amino acid residues were forming hydrogen bonding with L-mimosine, and Gly 70, Asn 170, Thr 216, Ala 237, and Gly 236 were forming hydrophobic interaction (Figures 7(c) and 7(d)). There were a greater number of hydrogen bonding and hydrophobic interaction between cefotaxime and the protein than between mimoine and the protein which suggest a greater binding affinity of cefotaxime with *Klebsiella pneumoniae*beta-lactamase. There was a greater binding affinity of antibiotics with almost all selected bacterial proteins, except *Bacillus cereus* metallothiol transferase; however, the binding of L-mimosine was strong with the active sites of all the targeted protein. This suggests L-mimosine is likely to be a potent antibacterial compound.

## 4. Conclusion

The study revealed that the phytochemicals in an ethyl acetate extract of *Mimosa pudica* have potent antibacterial activity as well as significant antioxidant efficiency. Although the parameters chosen for this research were not disease-specific, the assessment of antioxidant potentials can be used as a benchmark for the application* of Mimosa pudica* for reactive oxygen species-mediated diseases. In addition, the minimal drying loss observed during the physiochemical analysis could be correlated with the extract's potential to prevent itself from eroding, while performing in silico molecular docking of L-mimosine against selected bacterial protein showcased noteworthy antibiotic binding affinities. This shows that L-mimosine could be a potent antibacterial compound and a compound of interest for novel antibacterial drug discovery to combat antibiotic resistance.

## Figures and Tables

**Table 1:** Phytochemicals screening of ethyl acetate extract of *Mimosa pudica* leaf.

S. no.	Secondary metabolites	Phytochemical test	Results
1.	Carbohydrates	Molisch's	Presence
		Fehling	
		Barford	
		Benedict	

2.	Protein and amino acid	Biuret	Absence
		Ninhydrin	

3.	Phytosterol and triterpenoids	Liebermann	Absence
		Salkowski	

4.	Tannin	Ferric chloride	Absence
		Gelatin	
		Lead acetate	

5.	Flavonoids	Shinoda	Presence
		Alkaline reagent	

6.	Alkaloids	Mayer	Presence
		Wagner	
		Hager	

7.	Fixed oil and fats	Spot	Absence

8.	Saponin	Foam test	Absence

9.	Glycosides	Keller Killani	Presence

**Table 2:** Physicochemical analysis of the crude powder of *Mimosa pudica* leaves.

Physicochemical parameters	Extraction value (%)	Total ash content (%)	Loss on drying (%)
Obtained value	8.966	7.67	2.30

**Table 3:** Total phenolic content of *Mimosa pudica* extract.

Sample	Total phenolic content (mg GAE/g) (DE)
Ethyl acetate extract of *Mimosa pudica* (EAMP)	418.640 ± 0.018
Quercetin	—

**Table 4:** Total flavonoid content of *Mimosa pudica* extract.

Sample	Total flavonoid content (mg QE/g) (DE)
Ethyl acetate extract of *Mimosa pudica* (EAMP)	14.126 ± 0.021
Quercetin	—

**Table 5:** Antioxidant potency (IC_50_ *µ*g/ml) of *Mimosa pudica* dry extract.

Sample	DPPH free radical scavenging assay (IC_50_ *µ*g/ml)
Ethyl acetate extract of *Mimosa pudica* (EAMP)	158.95 ± 1.12
Quercetin	5.23 ± 0.38

**Table 6:** Interpretation of zone of inhibition given by *Mimosa pudica* against the selected bacterial strains.

Bacteria	Zone of inhibition (in mm) given by plant extracts of different concentrations				
	12.5 mg/ml	25 mg/ml	50 mg/ml	100 mg/ml	200 mg/ml
*Staphylococcus aureus*	7	8	10	12	15
*Bacillus cereus*	15	16	20	21	22
*Escherichia coli*	12	13	14	15	17
*Klebsiella pneumoniae*	11	12	13	14	16

**Table 7:** Interpretation of zones of inhibition given by positive and negative control against the selected bacterial strains.

Bacteria	ZOI of positive control (in mm)			Negative control
	Gentamicin	Cefotaxime	Clotrimazole	Ethyl acetate
*Staphylococcus aureus*	16	20	20	—
*Bacillus cereus*	20	13	15	—
*Escherichia coli*	18	21	—	—
*Klebsiella pneumoniae*	15	18	14	—

**Table 8:** Binding affinity shown by antibiotic and L-mimosine against the protein of bacteria.

Bacteria	Protein	Ligand	Binding affinity (kcal/mol)
*Escherichia coli*	DNA gyrase subunit B	Clorobiocin	−9.2
		L-Mimosine	−5.7

*Staphylococcus aureus*	DNA gyrase subunit B	Novobiocin	−8.2
		L-Mimosine	−6.1

*Bacillus cereus*	Metallothiol transferase	Fosfomycin	−4.3
		L-Mimosine	−5.2

*Klebsiella pneumonia*	Beta-lactamase	Cefotaxime	−7.5
		L-Mimosine	−6.1

## Data Availability

The data used to support the findings of this study are available from the corresponding author and first author upon request.
